# 1138. Optimal Duration of Systemic Corticosteroids Use in COVID-19 Treatment: A Meta-analysis

**DOI:** 10.1093/ofid/ofac492.976

**Published:** 2022-12-15

**Authors:** Nyein Yu, Paddy Ssentongo, David Ingram, Catharine I Paules

**Affiliations:** Penn State Health Milton S. Hershey Medical Center, Hershey, Pennsylvania; Penn State College of Medicine, Hershey, Pennsylvania; Penn State Health, Milton S. Hershey Medical Center, Hershey, Pennsylvania; Penn State Health Milton S. Hershey Medical Center, Hershey, Pennsylvania

## Abstract

**Background:**

Corticosteroids confer a survival benefit in hospitalized COVID-19 patients requiring oxygen, but optimal treatment duration remains uncertain. The goal of this meta-analysis is to determine the optimal duration of corticosteroids in the treatment of severe COVID-19.

**Figure:**
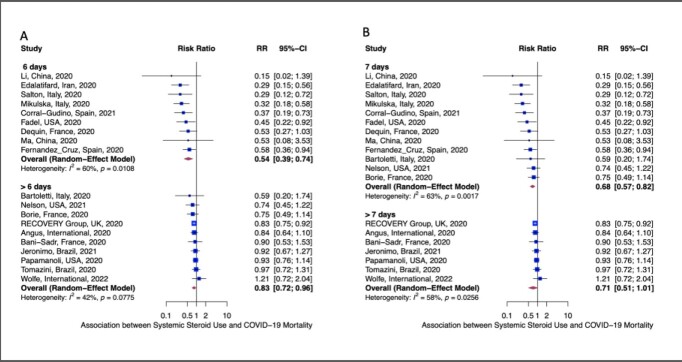
The effect of systemic steroids on mortality stratified by the duration of treatment in days.

The greatest survival benefit was observed for the treatment duration of up to 6 days (A). No survival benefit was observed beyond 7 days of treatment (B).

**Methods:**

Electronic databases (MEDLINE, Scopus, Cochrane Central Register of Controlled Trials, Cochrane Database of Systematic Reviews, the World Health Organization Global Literature on Coronavirus Disease, CoronaCentral, and Web of Science) and trial registries were searched to March 9, 2022, for randomized controlled trials and observational cohort studies reporting corticosteroid versus no corticosteroid treatment in hospitalized COVID-19 patients. Risk of bias was assessed using the Cochrane Risk of Bias tool (randomized controlled trials) or the Newcastle-Ottawa Scale (observational studies). We estimated the effect of corticosteroids on mortality by random-effects meta-analyses using the generic inverse variance method. Subgroup analyses and meta-analyses were conducted to assess the optimal duration of corticosteroid treatment.

**Results:**

We identified 28 eligible studies consisting of 13,404 hospitalized COVID-19 patients. Median age was 62 years (interquartile range: 59 – 67), and 65% were male. Eight randomized controlled trials and 20 observational studies were included in the meta-analysis of mortality, which suggested a protective association with corticosteroid therapy (risk ratio, 0.75; 95% CI: 0.64; 0.87). Pooled analysis of 19 studies showed the greatest survival benefit for a treatment duration up to 6 days (risk ratio, 0.54; 95% CI, 0.39; 0.74). Survival benefit was 0.68 (95% CI, 0.57; 0.82) up to 7 days, and no survival benefit was observed beyond 7 days of treatment (risk ratio, 0.71; 95% C: 0.51; 1.01).

**Conclusion:**

In this meta-analysis, the optimal duration of corticosteroid treatment for hospitalized COVID-19 patients was up to 6 days, with no additional survival benefit with > 7 days of treatment. Future analyses should stratify survival benefit by baseline disease severity to see if subgroups of patients derive greater benefit from longer courses of steroids.

**Disclosures:**

**All Authors**: No reported disclosures.

